# Three cases of type-1 complex regional pain syndrome after elective total hip replacement

**DOI:** 10.1051/sicotj/2017038

**Published:** 2017-09-05

**Authors:** Gerardo Zanotti, Pablo Ariel Slullitel, Fernando Martín Comba, Martín Alejandro Buttaro, Francisco Piccaluga

**Affiliations:** 1 Hip Surgery Unit, Institute of Orthopaedics “Carlos E. Ottolenghi”, Italian Hospital of Buenos Aires 4247 Potosí St. C1199ACK Buenos Aires Argentina

**Keywords:** Total hip arthroplasty, Complex regional pain syndrome, Painful total hip replacement, Complications of hip replacement surgery

## Abstract

Complex regional pain syndrome (CRPS) constitutes an atypical cause of pain after orthopaedic procedures. To our knowledge, there is a paucity of literature reporting this syndrome after total hip arthroplasty (THR), since only two case reports have been published. We thenceforth describe the clinical outcome of three cases of type-1 CRPS developed after elective THR, two of them initially diagnosed with secondary osteoarthritis whereas the remaining one presented a sequel of a failed osteosynthesis that required conversion to THR. Remission of disease was found at an average seven months (range: 4–9). Medical treatment involved a combined therapy of pain management, bisphosphonates and intense physical therapy. One patient was additionally treated with a corticosteroid blockade of his right sympathetic lumbar ganglia. None of the patients required surgical treatment. At final follow-up, physical examinations and imaging were negative for disease.

## Introduction

Relentless postoperative pain remains one of the major causes of patient dissatisfaction after primary total hip replacement (THR) [1]. In most cases, high preoperative levels of pain and poor subjective function have proven to be useful predictors of catastrophic clinical outcomes in patients undergoing total joint arthroplasty [2]. However, there are some unusual cases in which the genesis of postoperative pain cannot be easily interpreted by the surgeon, usually in the context of clinico-radiologic dissociation. Complex regional pain syndrome (CRPS) constitutes an atypical cause of pain after orthopaedic procedures. It has been generally related to painful conservative or surgical distal radius fracture treatment, under the term of “Sudeck syndrome” [3]. To our knowledge, there is a paucity of literature reporting this syndrome after THR, since only two case reports have been published [4, 5]. We thenceforth describe the clinical outcome of three cases of CRPS developed after elective THR, which share similarities and differences with the cases already reported.

## Case report 1

A 26-year-old male (BMI: 28.5) with bilateral dysplastic hip osteoarthritis, graded as Hartofilakidis A [6], underwent a one-stage bilateral sequential cementless THR (Corail^TM^, Depuy, Leeds, UK) with a fourth generation ceramic on ceramic bearing surface. As the patient complained of more excruciating symptoms on his left hip, this one was the first to be operated upon. The preoperative modified Harris Hip Score (mHHS) [7] was 55 points for the right hip and 50 for the left one. A posterolateral approach with the patient positioned in lateral decubitus was done, under epidural hypotensive anaesthesia, with a single preoperative dose of intravenous tranexamic acid and cephazoline administration. Besides preoperative planning and in order to calculate limb lengthening, the Woolson method was used with a Steinman pin inserted proximal to the acetabulum, as a stable pelvic reference point [8]. No acetabular reconstruction with bone graft was necessary.

Both the surgical procedure and the following 24 h of the postoperative course were uneventful. Following the first walking-assisted session with the physical therapist, the patient complained of a sudden incomplete loss of dorsal flexion of his left foot with no other associated symptom, categorized as grade 2 of the Medical Research Council (MRC) classification [9]. The right lower extremity remained asymptomatic. Initially interpreted as a lateral popliteal nerve (LPN) palsy, the patient was discharged at the fifth postoperative day. However, at the 10th postoperative day the patient was readmitted at the institution’s emergency service due to unbearable pain referred on the left foot and ankle, without a history of trauma. Defined by the patient as 10 points from the Visual Analogue Scale (VAS), pain could be alleviated neither by intravenous non-steroidal anti-inflammatories nor by narcotic drugs. The affected region presented marked subcutaneous oedema with diffuse paraesthesia and trophic alterations of the skin and appendages. Additionally, the patient presented a partial loss of the complete muscular function of the leg, not only affecting the LPN’s innervated territory. All these signs caused depression and anxiety to arise as conjoined symptoms.

**Figure 1.**
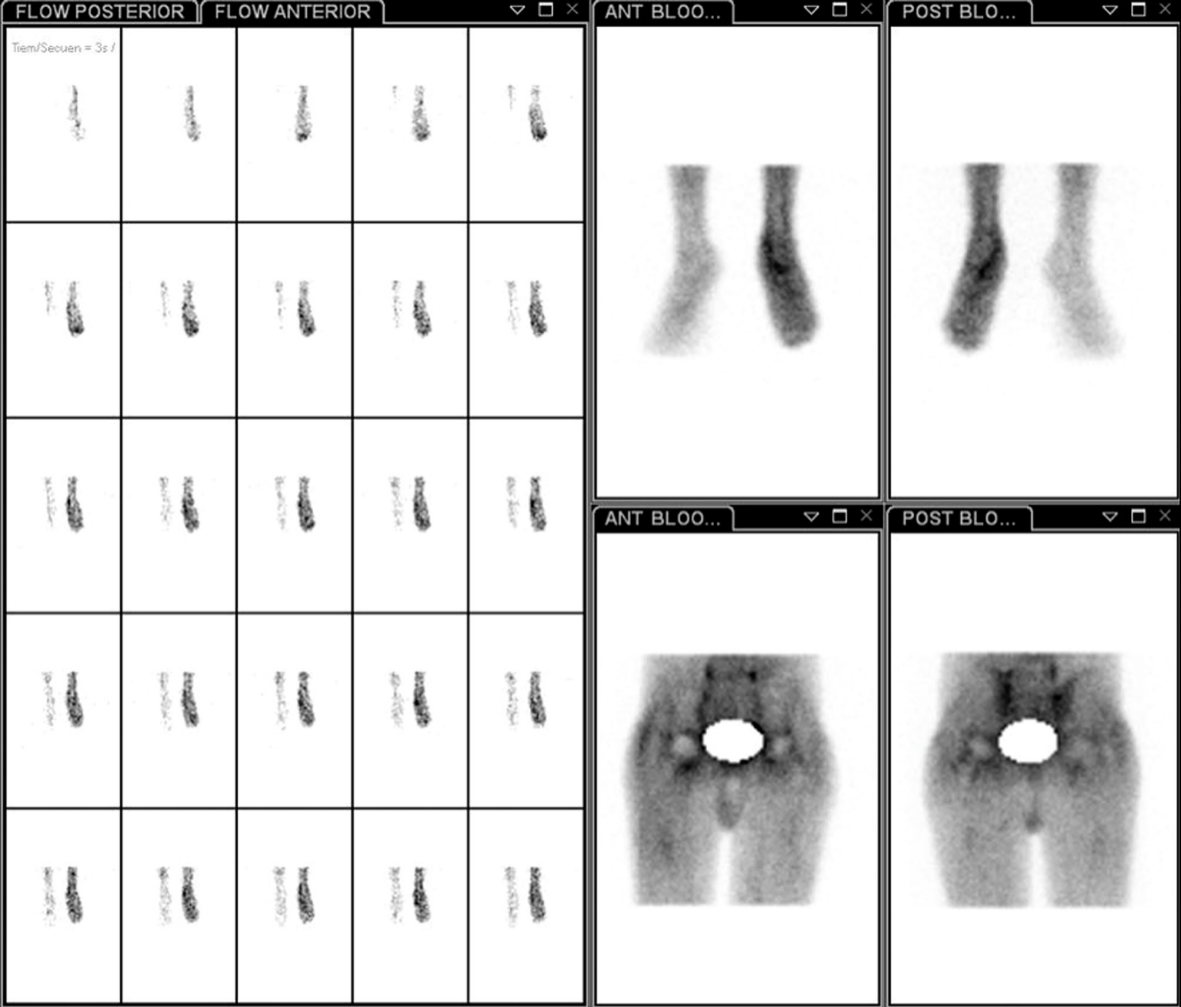
Resumed images of a triple-phase scintigraphy of a 26-year old male with a history bilateral hip dysplasia who underwent a one-stage bilateral sequential cementless total hip arthroplasty two months previously, showing evidence of increased hyperperfusion and bone turnover in the left foot and ankle at the second and third phases, compatible with Sudeck syndrome.

Under a multidisciplinary approach, the patient was further studied with conventional radiographs of the foot and ankle, which evidenced subtle osteoporotic changes. Bone densitometry confirmed these findings by displaying 21% more density on the right foot when compared to the left one (1.385 g/cm^2^ vs. 1.089 g/cm^2^). Bone scintigraphy, done two months postoperatively, showed an increased diffuse bone turnover in the left foot and ankle at the second and third phases (1 and 3 h after the isotope injection, respectively), suggesting a moderately chronic disease (Figure 1). Additionally, the bone scan did not reveal any abnormality on either hip.

Given these findings together with allodynia, the presumptive diagnosis of type-1 Sudeck disease was made. Hence, the Pain Management Team, which involved an anaesthesiologist, an endocrinologist, a psychologist, a physical therapist and two physician assistants, advised medical treatment with: 10 mg of oxycodone once a day; pregabalin 75 mg every 12 h and 80 mg of 1,2-dehydrocortisol once daily for seven days. Moreover, 1 mg of clonazepam once a day was indicated as chronic medication to treat depression. At nine months follow-up, symptoms and allodynia disappeared, with the patient mobilizing without walking aids. On VAS, his left foot pain was 0/10. At latest three years follow-up, mHHS was 95 and 90 points for the right and left hips, respectively.

## Case Report 2

A 28-year old male (BMI: 23) presented with secondary hip osteoarthritis due to a progressive Legg-Perthes disease on his right hip. Preoperative mHHS was 48 points. An uncemented THR (Corail^TM^, Depuy, Leeds, UK) with a fourth generation ceramic on ceramic bearing surface was performed. The surgical protocol was similar to Case 1, using epidural anaesthesia in the lateral decubitus position, through a posterolateral approach. Postoperative course was normal until the second postoperative day, on which the patient presented with a grade 3 [9] loss of motor power on his right foot, along the LPN distribution. Concomitant symptoms consisted of hyperalgesia, local sweating and trophic alterations of the skin on the same foot and ankle, similar to Case 1. Pain VAS, reported as 10/10, triggered a hyper-anxiety disorder that affected sleep, which could not be counterbalanced by commonly used benzodiazepines.

**Figure 2.**
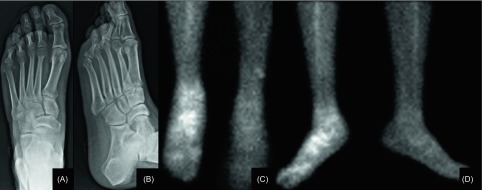
(A) Anteroposterior and (B) oblique right foot’s X-ray of 28-year old male, 15 days after he underwent a total hip replacement due to an advanced Legg-Perthes disease on his right hip, showing minimal osteoporotic changes on the right foot and ankle, especially at the tarsus and metatarsophalangeal joints. (C) Third-phase anteroposterior and (D) lateral bone scan images of both legs and feet of the same patient, displaying increased isotope accumulation along the right foot.

As shown in Figure 2, radiographs demonstrated minimal osteoporotic changes on the right foot and ankle. Triple-phase bone scintigraphy performed during hospital stay showed hyperperfusion of the right foot on both the vascular and late phases of the right foot (Figure 2) as well as around the acetabular and femoral components, considered as acute reparative changes following THR. Since symptoms persisted for a one-month period, a new bone scan was done. In this case, increased tracer accumulations could be observed in the region of the talus and metatarsus on the osseous phase, compatible with reflex sympathetic dystrophy syndrome (RSDS).

Medical treatment was similar to Case 1. However, because of signs of chronic disturbance on the sympathetic nervous system, which involved persistent augmented temperature on the right foot, swelling and allodynia, a computed tomography (CT)-guided sympathetic blockade of the right sympathetic trunk was done, using 4 mL hyperbaric Bupivacaine and 8 mg Dexamethasone. Symptoms decreased progressively until eight months after the blockade, on which pain ceased completely. At six years follow-up, mHHS was 92 points, with the patient doing all activities of daily living and practising low-impact sports.

## Case Report 3

**Figure 3.**
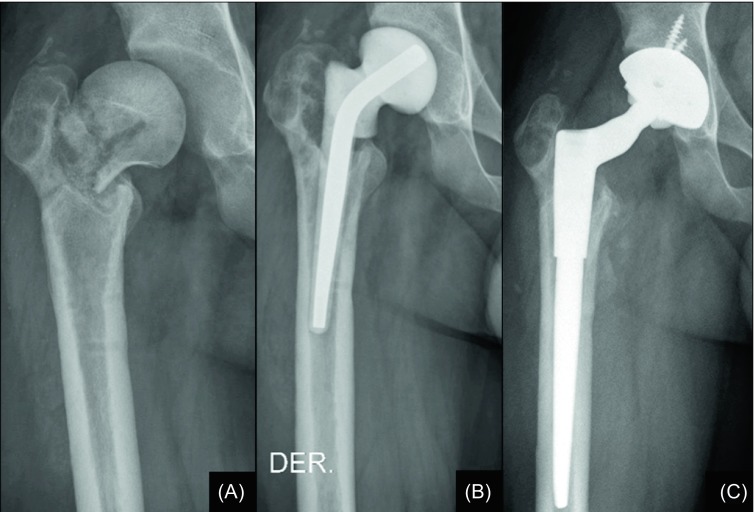
(A) Anteroposterior right hip radiograph of a 25-year old male with sequelae of a traumatic basicervical right hip fracture treated at another institution one year earlier, evidencing signs of non-union. (B) Anteroposterior X-ray view of the same patient after treatment with an antibiotic-impregnated cement spacer, as part of a two-stage protocol revision surgery. (C) Radiographic image of the same hip eight weeks later, following reimplantation with a distally fixed uncemented stem and a cementless acetabular component.

A 25-year old male with a history of a traumatic basicervical right hip fracture evolved with an ipsilateral infected non-union, diagnosed through a CT-guided biopsy. Given the proximal femoral metaphyseal osseous defect, in the context of bone chronic infection, a two-stage revision THR was planned (Figure 3). Forty-five days after reimplantation with a cementless acetabular cup and a distally fixed uncemented femoral stem (ZMR^TM^ Zimmer, Warsaw, Indiana, USA), the patient was received at the institution’s emergency service due to diffuse intolerable pain on the ipsilateral lower extremity, starting from the knee down to the metatarsophalangeal joints. Pain was not responsive to intravenous painkillers. Subcutaneous swelling was remarkable as well as skin colour changes with disappearance of the skin’s wrinkles all along the right foot and ankle. Normal laboratory parameters and a closed wound suggested absence of reinfection.

**Figure 4.**
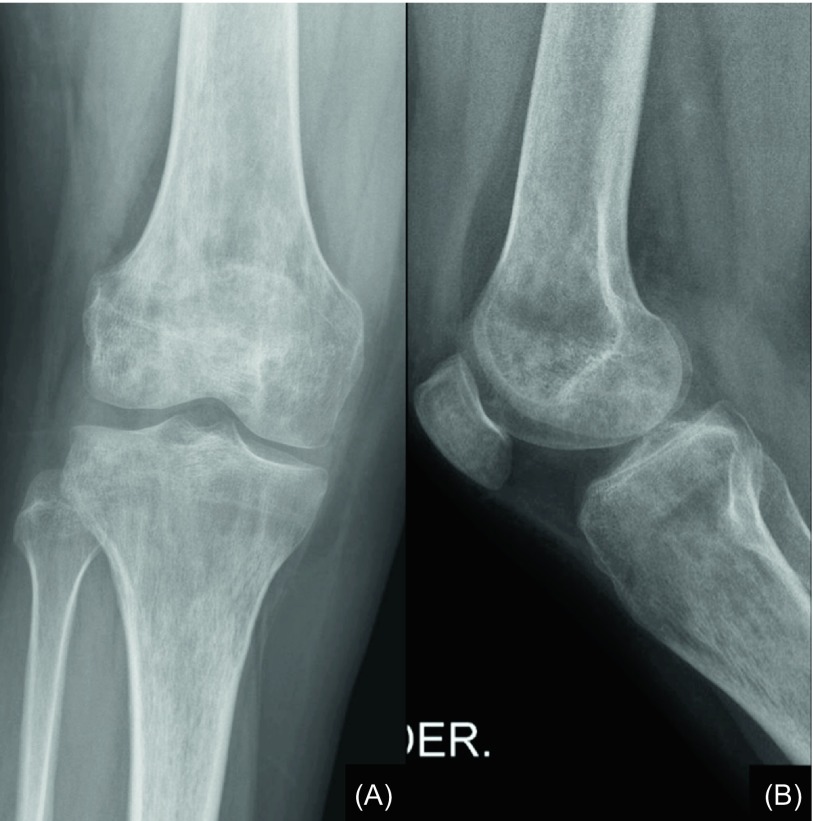
(A) Anteroposterior and (B) lateral radiographs of the patient mentioned on Figure 3 at a one-month period after the performance of the reimplantation, presenting mottled osteoporotic changes all along the distal femur and proximal tibia.

**Figure 5.**
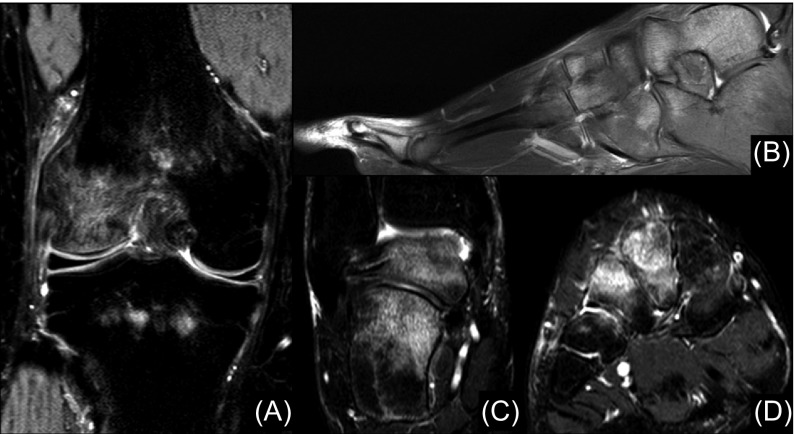
(A) T2 fat suppressed-coronal image of the right knee of the patient cited before (Figures 3 and 4) revealing diffuse intraosseous oedema, especially in the lateral femoral condyle. (B) T2 sagittal image of the patient’s right foot showing disperse bone marrow oedema of both hindfoot bones, tarsal bones and proximal phalanxes. T2 fat suppressed-coronal images of the patient’s (C) right hindfoot and (D) midfoot, with marked oedema inside the talus, calcaneus and 2nd and 3rd cuneiform bones.

Radiographs revealed diffuse, stippled osteoporotic changes in the right knee (Figure 4) and foot and ankle. Accordingly, magnetic resonance imaging (MRI) of the right lower extremity showed intraosseous oedema in the distal femoral lateral epicondyle, proximal tibial metaphysis, talus, calcaneus, tarsal bones and metatarsophalangeal joints (Figure 5). Dual-energy X-ray absorptiometry correlated that oedema with lesser focal mineral density and bone scanning indicated hyperperfusion on the mentioned areas.

The Pain Management Team advised medical treatment with bisphosphonates (alendronate 7.5 mg intravenously daily for three days) and 10 mg of oxycodone once a day; pregabalin 75 mg every 12 h and 80 mg of 1,2-dehydrocortisol once daily for seven days. The patient was pain-free at four months postoperatively, walking without aids. At four years follow-up, mHHS score was 88, without signs of loosening or reinfection.

## Discussion

Since its first description in 1916 [10], CRPS has been rarely described after hip surgery and can easily be ignored by the inexperienced physician [4, 5, 11]. Patman et al. [12] reported 113 cases of post-traumatic pain syndrome of which only 18 had their diagnosis made by the treating physician. Causalgia, post-traumatic pain syndrome, reflex sympathetic dystrophy syndrome (RSDS), Sudeck syndrome and algodystrophy are many of the CRPS’s synonyms that had been used over the past decades. This varied terminology seems to be correlated with an under-reported rate following painful hip arthroplasties, usually referred to as idiopathic pain [13]. In this scenario, we aimed to describe three patients with Sudeck dystrophy developing after an elective THR and review the literature available on this topic.

In 1993, the International Association for the Study of Pain (IASP) characterized this syndrome by pain, which is disproportionate to its trigger, associated with sensitive, motor and/or autonomic symptoms generated by any noxious stimulus [14]. Its diagnosis is largely clinical and is a diagnosis of exclusion [15]. Nevertheless, symptoms are sometimes not so evident and CRPS’s identification may remain unobserved. In the described cases, the suspicion of CRPS was developed by the primary surgeon and both definite diagnosis and treatment were made through a multidisciplinary approach. The IASP has described two different types of CRPS: type 1, in which symptoms occur without any previous nerve injury and type 2, in which symptoms are triggered after a specific nerve lesion. Although Cases 1 and 2 had been described with a loss of motor power similar to that of LPN palsy, both patients referred their motor-sensitive symptoms after an initial 24- and 48-h symptom-free period, respectively. In this sense, both patients had already initiated their rehabilitation protocol walking with full weight bearing with crutches before CRPS established.

Case 3 presented with incessant pain starting from the ipsilateral knee. Sudeck syndrome seems to have a flair for knee localization. On the one hand, the knee has historically been a typical symptomatic spot of idiopathic CRPS [16, 17], producing pain and locking; on the other, CRPS has been thoroughly described after total knee replacement (TKR). Ritter [18] referred six cases of causalgia out of 439 posterior cruciate-retaining total condylar knee arthroplasties, considering it a major cause of moderate to severe pain that should be contemplated in every algorithm of painful TKR. Katz et al. [19] described five patients out of 662 primary TKR with RSDS that settled during the postoperative course, four of whom revealed manifest limitation of flexion requiring manipulation under anaesthesia, in addition to excessive pain and cutaneous hypersensitivity. In their series, sympathetic blockade was key for both diagnosis and treatment as well. The authors alleged that classic RSDS findings of objective vasomotor alterations and radiographic osteopenia might be tough to decode in patients with TKR.

Hossain and Andrew [4] described a case of RSDS after primary THR in a diabetic patient. Non-mechanical pain was unremitting with paraesthesia referred to the ipsilateral foot; but, unlike the cases we reported, there was no affection of muscle power or neurological status. Although most radiological features were normal, three-phase bone scanning revealed massive isotope uptake affecting the ipsilateral foot and ankle along the phase III, similarly to our cases. In an analogous case, Mittal et al. [5] highlighted the role of Tc-99m bone scan to differentiate CRPS from other causes of unidentified pain after THR. Like Katz et al. [19], the authors used sympathetic blockade to confirm the diagnosis and treat the condition.

In this sense, complementary imaging offers an imperative diagnostic tool. Bone scintigraphy appears as the most sensitive imaging technique for the diagnosis of CRPS, especially within the first 20–26 weeks of onset [20]. The vast majority of positive results are found at the interval (phase II) and late (phase III) phases, in which tracer accumulation correlates with clinical and roentgenologic findings [21]. Nonetheless, sometimes scintigraphy is useful and even more sensitive for diagnosis and assessment of the therapeutic response in the absence of evident radiographic demineralization [22]. Usually, acute, patchy osteoporotic changes can be seen on conventional radiographs of the ipsilateral knee and/or foot and ankle or at densitometry examinations, which is concomitant with intense focal bone marrow oedema that can be appreciated on MRI [23]. Our described cases’ imaging matches these reported findings, which would lose specificity in the absence of excruciating pain.

Treatment options are varied and most reports indicate that quick commencement of a precise therapy leads to an earlier improvement [3, 24]. In our cases, symptoms ceased at an average seven months (range: 4–9). A multidisciplinary approach is vital since multiple drug therapies are used. The psychiatric medical treatment is not less important [25], since most of these patients seem to have lower baseline levels of stress and depression. Bisphosphonates seem to play a role when osteopenia is massive [26] whereas corticosteroids, non-steroidal anti-inflammatory drugs as well as gabapentin-related painkillers have also proven useful for pain alleviation [27, 28]. The efficacy of sympathetic blockades remains inconclusive due to scarcity of published evidence [29]. However, many authors [4, 19, 30] have encouraged the use of these blockades for CRPS following hip, knee or lumbar surgeries. Additionally, we have obtained excellent results in one of the reported cases (Case 2) in which signs of autonomous nervous system’s affection were severe, with augmented local temperature, massive swelling and allodynia.

CRPS should be considered in the diagnostic algorithm of painful THR, especially when it is disproportionate to a noxious stimulus. When clinical symptoms are not so evident, complementary imaging, chiefly with triple-phase bone scan, plays a major role in diagnosis. The faster the treatment is initiated, the better the clinical outcome.

## Conflict of interest

Each author certifies that he or she has no commercial associations (e.g., consultancies, stock ownership, equity interest, and patent/licensing arrangements, etc.) that might pose a conflict of interest in connection with the submitted paper.

GZ, PS, FC, MB and FP certify that they have no financial conflict of interest (e.g., consultancies, stock ownership, equity interest, patent/licensing arrangements, etc.) in connection with this article.
